# Inflammation, Oxidative Stress, and Antioxidants Contribute to Selected Sleep Quality and Cardiometabolic Health Relationships: A Cross-Sectional Study

**DOI:** 10.1155/2015/824589

**Published:** 2015-10-19

**Authors:** Thirumagal Kanagasabai, Chris I. Ardern

**Affiliations:** School of Kinesiology and Health Science, York University, Toronto, ON, Canada M3J 1P3

## Abstract

Sleep is vital for cardiometabolic health, but a societal shift toward poor sleep is a prominent feature of many modern cultures. Concurrently, factors such as diet and lifestyle have also changed and may mediate the relationship between sleep quality and cardiometabolic health. Objectives were to explore (1) the interrelationship and (2) mediating effect of inflammation, oxidative stress, and antioxidants on sleep quality and cardiometabolic health. Cross-sectional data from the US National Health and Nutritional Examination Survey 2005-06 (≥20 y; *N* = 2,072) was used. Cardiometabolic health was defined as per the Joint Interim Statement; overall sleep quality was determined from six sleep habits and categorized as good, fair, poor, and very poor. Fair quality sleepers had optimal inflammation, oxidative stress, and antioxidant levels. Inflammation was above the current clinical reference range across all sleep quality categories, while oxidative stress was only within the clinical reference range for fair sleep quality. Selected sleep quality-cardiometabolic health relationships were mediated by inflammation, oxidative stress, and antioxidants and were moderated by sex. Our results provide initial evidence of a potential role for inflammation, oxidative stress, and antioxidants in the pathway between poor sleep quality-cardiometabolic decline. Further prospective research is needed to confirm our results.

## 1. Introduction

Sleep is a vital human process needed for optimal immune, cardiometabolic, and cognitive health [1]. However, a societal trend toward less sleep and poorer quality sleep is a common feature in many developed countries [2, 3]. Whether sleep duration or quality is more important for health remains unclear, but some research suggests they modify each other's associations with health [4]. After adjusting for sleep duration, significant associations between sleep quality and cardiometabolic health were found in several population studies [4–6].

However, few studies have explored the association of sleep quality with inflammation, oxidative stress, and antioxidants levels [7, 8]. Observational studies support a modest-to-strong correlation between sleep quality measures and inflammation (i.e., C-Reactive Protein (CRP)) [9, 10], but the relationship may be sex-specific. For instance, Liu et al. [10] found that poor quality sleep was significantly associated with CRP in women, but not men. Additionally, the role of oxidative stress in sleep quality as well as the beneficial effect of antioxidant therapy has been demonstrated in obstructive sleep apnea patients [11]. Furthermore, the relationship between these factors (i.e., inflammation, oxidative stress, and antioxidants) and cardiometabolic health is also known and collectively suggests that abnormal levels of inflammation, oxidative stress, and insufficient antioxidants are associated with many age-related diseases, including diabetes, cardiovascular disease, and cancer [12]. Previously, we explored the interrelationship and mediating effect of inflammation (i.e., CRP), oxidative stress (i.e., *γ*-glutamyl transferase (GGT)), and antioxidants (i.e., bilirubin, carotenoids, uric acid, and vitamins A, C, D, and E) on* sleep duration* and cardiometabolic health [13]. However, the role of* sleep quality* within this context has not yet been explored. Thus, the purpose of this study is to (i) explore the interrelationship between sleep quality and inflammation (CRP) [14], oxidative stress (GGT) [15], and antioxidants (bilirubin, carotenoids, uric acid, and vitamins A, C, D, and E) [16, 17] and (ii) quantify the indirect mediating effect of these factors on the sleep quality-cardiometabolic health relationships in free-living adults.

## 2. Methods

### 2.1. Participants

Data for this analysis was drawn from the US National Health and Nutrition Examination Survey (NHANES) [20]. NHANES is a series of nationally representative cross-sectional studies designed to assess the health and nutritional status of the US noninstitutionalized civilian population of all ages and ethnicities. Approximately 10,000 persons are sampled biannually, and data are collected from personal interviews, standardized physical examinations, and laboratory samples. NHANES 2005-2006 cycle, which had an initial sample of 10,348 individuals, was used in this study. Subsequent exclusions were made for age (<20 y: *N* = 5,369), pregnancy (*N* = 336), missing MetS components (*N*_waist  circumference_ = 455, *N*_triglyceride_ = 196, *N*_blood  pressure_ = 79, *N*_fasting  plasma  glucose_ = 1,826, and *N*_HDL  cholesterol_ = 0), and missing sleep quality variables (*N* = 15) for a final analytic sample of 2,072.

### 2.2. Metabolic Syndrome and Cardiometabolic Health

The Joint Interim Statement was used to define metabolic syndrome (MetS) [≥3 of elevated waist circumference: men (≥102 cm) and women (≥88 cm); elevated triglycerides or medication: ≥1.69 mM; low HDL cholesterol or medication: men (<1.04 mM) and women (<1.29 mM); elevated blood pressure or medication: systolic (≥130 mmHg) and/or diastolic (≥85 mmHg); and elevated fasting plasma glucose or medication use (≥5.6 mM)] [21]. These criteria were summed to determine the number of MetS components [0, 1, 2, 3, 4, 5]. Cardiometabolic health markers assessed were the individual MetS components included in the Joint Interim Statement.

### 2.3. Sleep Quality

The Sleep Disorders Questionnaire was administered to participants aged ≥16 y (limited to adults ≥20 y for our analysis), who reported their typical sleep habits for the past month [20]. This questionnaire contains items from two previously validated sleep questionnaires [22]. Overall sleep quality was determined from six questions: “How often did you have trouble falling asleep?”; “How often did you wake up during night and had trouble getting back to sleep?”; “How often did you wake up too early in morning and were unable to get back to sleep?”; “How often did you feel unrested during the day, no matter how many hours of sleep you had?”; “How often did you feel excessively or overly sleepy during day?”; and, “How often did you not get enough sleep?” Participants' responses to each question [0 = never; 1 = rarely (1 time a month); 2 = sometimes (2–4 times a month); 3 = often (5–15 times a month); and 4 = almost always (16–30 times a month)] were summed to obtain an index of overall sleep quality. The sleep quality score was subsequently categorized as good (0 to <3); fair (3 to <7); poor (7 to <12); and very poor (≥12 to 24) [3].

### 2.4. Mediators and Population Descriptors

Mediators considered in this study were CRP, GGT, bilirubin, carotenoids, uric acid, and vitamins A, C, D, and E and were obtained from laboratory files [23]. Blood samples were collected by certified phlebotomists, and we used the morning session's data which contained blood samples after an overnight fast [23]. Demographic variables used to describe the population include age (categorized as 20 to <40 y, 40 to <65 y, and ≥65 y), sex (men, women), ethnicity (Non-Hispanic White, Non-Hispanic Black, Mexican American, and Others), income (<$20,000, $20,000–44,999, and ≥$45,000), education (<high school, high school, and college), alcohol intake (0, <3, and ≥3 drinks per day), smoking history [current (if smoking now), past (if smoked ≥100 cigarettes in one's life but not current smoker), or never (if smoked <100 cigarettes in one's life)], and recreational physical activity (PA) adherence (none reported, <500 metabolic equivalent (MET)·min/wk and ≥500 MET·min/wk) [24, 25]. To estimate MET·min/wk for recreational PA adherence, we used NHANES suggested MET values, which were then categorized according to the PA guidelines for Americans [26].

### 2.5. Statistics

Mean and 95% confidence interval (CI) for continuous variables and frequency (%) and 95% CI for categorical variables were determined for each descriptor, by category of sleep quality. ANOVA (with* post hoc* Tukey's test) and *χ* ^2^ tests were used, as appropriate, to test for any differences in demographic and behavioral characteristics of sleep quality groups. The interrelationship between sleep quality and CRP, GGT, bilirubin, carotenoids, uric acid, and vitamins A, C, D, and E was determined and presented visually relative to the American Medical Association's clinical reference ranges [19]. Subsequent to this, the indirect mediation effect of CRP, GGT, bilirubin, carotenoids, uric acid, and vitamins A, C, D, and E on the sleep-cardiometabolic health relationship was estimated using (i) logistic regression for binary outcomes (i.e., MetS) and (ii) general linear models for the number of MetS components and individual cardiometabolic parameters [18].

**Figure 1:**
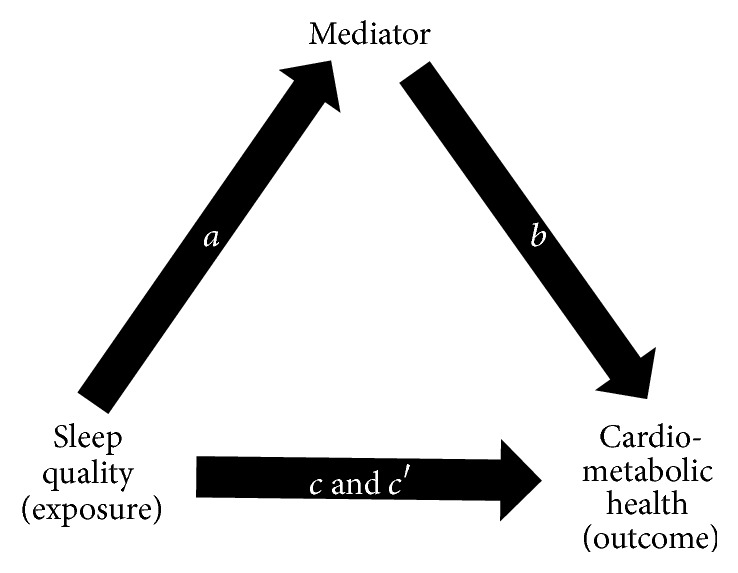
Multiple regression method of the indirect mediation model [18]. Path *a* indicates the path from sleep quality (exposure) to mediator (i.e., inflammation, oxidative stress, and antioxidant). Path *b* indicates the path from mediator to outcome (i.e., metabolic syndrome (MetS), number of MetS components, and individual MetS components) controlling for the mediator. Path *c* indicates the path from exposure to outcome. Path *c*′ indicates the path from exposure to outcome controlling for the mediator.

Through a series of regression analyses, indirect mediation helps explain the underlying relationship between an exposure and an outcome variable through a third (mediatory) variable [18] (Figure 1). These relationships are depicted by four pathways: regression between exposure and mediator (path *a*); regression between mediator and outcome while adjusting for the exposure (path *b*); regression between exposure and outcome (path *c*); and regression between exposure and outcome while adjusting for the mediator (path *c*′) [18]. Participants with a missing mediator variable were excluded from each regression analysis to ensure the products of *ab* and *c*-*c*′ were equivalent [18]. The product of ab was subsequently used to classify indirect effects as large (≥0.25), moderate (≥0.09), modest (≥0.01), and weak (<0.01) [18]. To detect a moderate indirect effect with 80% power, *n* = 105 participants were needed in each sleep quality category. Significance for indirect effect was tested with the Sobel and Joint Significance tests.

To ensure the representativeness of the data, the medical exam sample weight from the demographics data file was used to weight all analyses [20]. All analyses were conducted in SAS v9.3 (Cary, NC, U.S.A) with statistical significance set at an *α* of 0.05.

## 3. Results

Table 1 describes the characteristics of the US adult population, stratified by sleep quality categories. In general, sleep quality varied by sex, age, ethnicity, education, recreational PA, and smoking. Specifically, middle aged adults (40 to <65 y) tended to have very poor quality sleep, while older adults reported good quality sleep compared to other sleep quality categories. Very poor sleep quality was also more common among women, Non-Hispanic Whites, and current smokers. Compared to good and very poor quality sleepers, both fair and poor sleepers attained more recreational PA, half of whom, approximately, met the PA guidelines for Americans.

**Figure 2:**
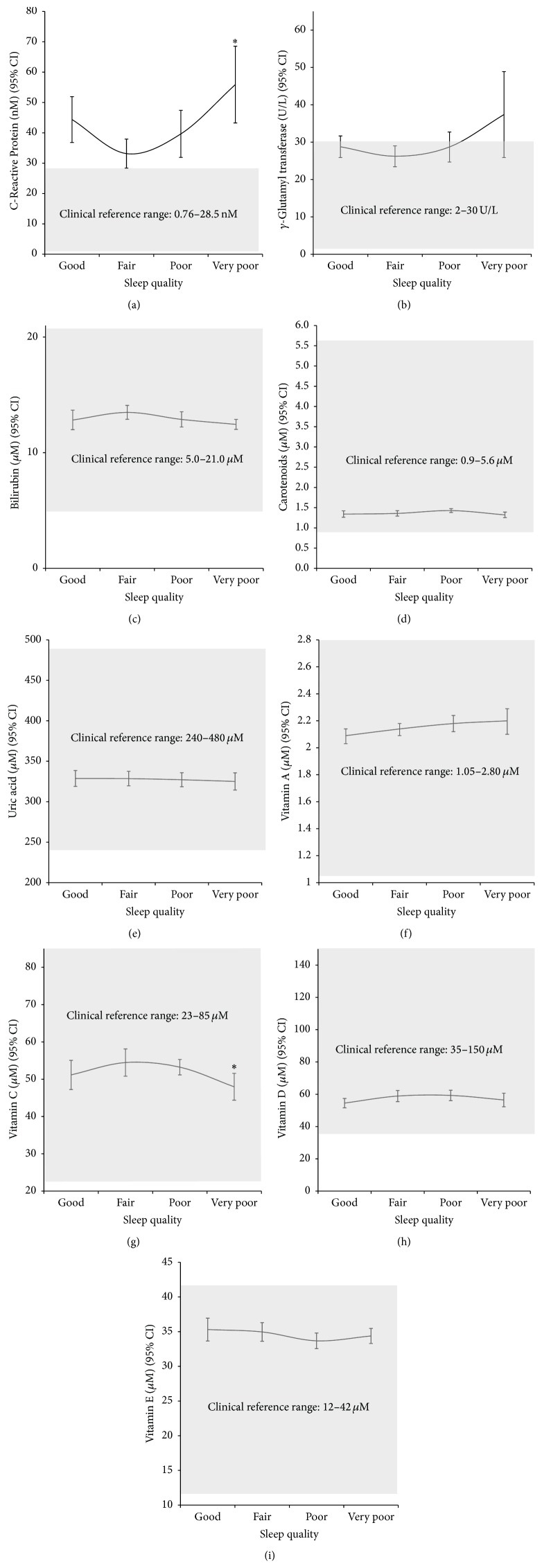
Interrelationship between sleep quality and inflammation (a), oxidative stress (b), and antioxidants ((c)–(i)). Gray shaded areas are clinical reference ranges [19]. ^*∗*^ *p* < 0.05 versus fair sleep quality.

Figure 2 illustrates the interrelationship between sleep quality and inflammation (a), oxidative stress (b), and antioxidants (c)–(i) with clinical reference ranges shaded in gray. Those reporting fair, but not good, quality of sleep had the optimal inflammation and oxidative stress profiles. However, inflammation was above the clinical reference range across all sleep quality categories. For GGT, all levels of sleep quality except those with very poor quality sleep had mean values within the clinical reference range. All antioxidants were within the clinical reference range and were generally optimal among fair or poor quality sleepers. CRP and vitamin C levels were statistically different for fair versus very poor quality sleep.

Overall, vitamins A and C were modest mediators of the sleep quality-MetS and sleep quality-number of MetS components relationships (Table 2). Further exploration of the indirect effect revealed these antioxidants were also moderate (≥0.09) mediators of the sleep quality-waist circumference and sleep quality-systolic blood pressure relationships (Table 3). Additionally, the sleep quality-waist circumference and sleep quality-diastolic blood pressure relationships were moderately mediated by CRP.

While some minor differences between men and women were observed for the sleep quality-MetS and sleep quality-number of MetS components relationships (men: Table 4; women: Table 5), the differences were larger for some individual components (men: Table 6; women: Table 7). In women, CRP, uric acid, and vitamin C were large mediators of the sleep quality-waist circumference relationship and moderately mediated by carotenoids and GGT. Uric acid was also a large mediator of the sleep quality-systolic blood pressure relationship in women, while CRP and carotenoids were moderate mediators. Finally, vitamin C was a moderate mediator of the sleep quality-diastolic blood pressure relationship in women.

## 4. Discussion

We found that very poor sleep quality was more common amongst women, Non-Hispanic Whites, current smokers, and middle aged adults (40 to <65 y), whereas older adults were more likely to report good quality sleep. Optimal inflammation and oxidative stress profiles were found amongst fair quality sleepers, while some antioxidants were also optimal amongst fair and poor quality sleepers. While most sleep quality-MetS or sleep quality-number of MetS components relationship were not significant, selected sleep quality-cardiometabolic health relationships were moderately mediated by CPR and vitamins A and C. Additionally, in women only, the indirect effects were moderate-to-large for CRP, GGT, carotenoids, uric acid, and vitamin C.

### 4.1. Inflammation, Oxidative Stress, and Antioxidants Profiles

Our finding of optimal inflammation, oxidative stress, and antioxidant levels amongst the fair or poor sleep quality categories was surprising, as we expected optimal levels amongst those with at least good sleep quality. To date, relatively few studies have investigated the interrelationship between inflammation, oxidative stress, and antioxidants with sleep quality [7, 8, 27–29]. In one such study (*n* = 24), adherence to a kiwi diet (2 kiwi/night for 4 weeks), a fruit rich in vitamins C and E and serotonin, improved sleep onset and duration [27]. Another similar study (*n* = 20) found that tart cherry juice, rich in vitamins A and C, improved sleep quality, suggesting increased melatonin levels as a possible mechanism for this effect [28]. However, neither of these studies measured serum antioxidant levels [27, 28]. In sleep disordered populations, elevated levels of inflammation and oxidative stress are common features [29]; and overall antioxidant capacity tends to decrease with age [30].

Another possible explanation is the definition of sleep quality, which is based on the six questions that were assigned equal weights [3]. In a previous analysis, we found that the odds of MetS varied between questions, with no significant relationship for getting enough sleep (OR: 1.06) and roughly twofold increases for trouble falling asleep, feeling overly sleepy, and waking up during the night, after adjusting for age, sex, ethnicity, education, income, smoking, recreational physical activity, and sleep duration (unpublished data). Therefore, future work may benefit from the development of a recalibrated (weighted index) of sleep quality.

### 4.2. Indirect Mediation Effect

Evaluating the indirect effect of inflammation, oxidative stress, and antioxidants on the relationship between sleep quality and cardiometabolic health was the second aim of our study. Although our overall findings were weak or modest for the sleep quality-MetS and sleep quality-number of MetS components relationships, moderate indirect effects by CPR and vitamin C on the sleep quality-waist circumference and vitamin A for sleep quality-systolic blood pressure relationships warrant discussion.

#### 4.2.1. Waist Circumference

Although the relationship between poor sleep quality and body weight is well known [31], little work has been done to examine the association with sleep quality. Positive associations between waist circumference and CRP or vitamin C have nonetheless been found [32, 33]. Evidence also suggests that sleep architecture may be altered in individuals with obesity; that is, rapid eye movement stage of sleep may occur earlier compared to normal weight individuals [34]. In sleep disordered populations, the prevalence of obesity is high; and both sleep quality and obesity have been independently linked to increased inflammation and decreased antioxidants [7, 14, 31]. For instance, a large Finnish study reported that CRP was elevated (≥9.52 nM) among men reporting frequent sleep disturbances, even after adjusting for BMI [35]. Vitamin C supplementation, on the other hand, improved endothelial function in patients with obstructive sleep apnea [36], a condition associated with increased bodyweight [15]. In rodents, the beneficial effects of vitamin C on weight gain and the absorption of lipids has been demonstrated [37, 38], and some more limited research suggests that vitamin C supplementation could help reduce adiposity through alterations in gene expression [39]. While the direct inflammatory and antioxidant mechanisms purported to influence the relationship between poor sleep quality and obesity remain unknown, our study provides initial evidence of a mediating role of CRP and vitamin C on the relationship between sleep quality and abdominal obesity.

#### 4.2.2. Systolic Blood Pressure

While evidence suggests vitamin C could improve endothelial function [36], we did not find that it significantly mediated the sleep quality-systolic blood pressure relationship; rather, we found vitamin A moderately mediated the sleep quality-systolic blood pressure relationship. In mice, deficiency in vitamin A decreased nonrapid eye movement (REM) sleep stages 3 and 4 (i.e., deep or slow wave sleep); and evidence in humans suggest that vitamin A has an important role in sleep homeostasis [40].

### 4.3. Sex-Stratified Indirect Mediation Effect

We found that CRP, GGT, carotenoids, uric acid, and vitamin C were moderate-to-large mediators of selected sleep-cardiometabolic health relationships in women only. Others have found similar sex differences between inflammation, oxidative stress, antioxidants, and cardiometabolic dysfunction [17, 41]. Increased systematic inflammation is a particular concern in aging women since poor sleep duration, poor sleep quality, lack of social interactions, and abdominal adiposity have all been associated with inflammation [42, 43]. Additionally, Okun et al. [44] demonstrated a link between inflammation and poor sleep quality, proposing that chronic low-grade inflammation as a result of poor sleep quality in early adulthood (i.e., 20s) may predispose women to inflammation-related diseases in middle-adulthood. Indeed, the relationship between inflammation, oxidative stress, antioxidants, and age-related diseases warrants further study in women. Changes in female sex hormone levels, for instance, are associated with longer sleep duration and poorer sleep quality [45], while risk of cardiometabolic disease [46] and adiposity [47] also changes with circulating testosterone, estradiol, and sex hormone binding globulin levels. Taken together, our findings suggest that strategies to improve dietary or sleep habits may be valuable for the cardiometabolic health of women.

### 4.4. Strengths and Limitations

There are several study limitations that warrant mention. First, given the preliminary and cross-sectional nature of our findings, future longitudinal studies are needed to evaluate evidence for a cause-and-effect relationship. Second, in applying our study exclusion criteria, our final analytic sample was limited to less than 50% of the initial adult sample. Third, since all sleep quality variables were self-reported, they are susceptible to recall and response biases. However, we are not aware of a comprehensive population-based dataset that contains objective measures of sleep quality along with other physical measures necessary for this research question [i.e., accelerometry, polysomnography, or electroencephalogram, serum biomarkers, and cardiometabolic health information]. We were also unable to study the effect of adipokines, such as leptin and adiponectin, which have been linked to poor sleep quality [48] and obesity [49]. Moreover, this work is limited to a select number of biomarkers for inflammation and oxidative stress. Future work would therefore benefit from the inclusion of interleukin-6, tumour necrosis factor-*α*, and malondialdehyde [50–52] in participants with diagnosed inflammatory diseases. Finally, since we used single measurements of exposure, outcome, and mediators, we are unable to account for potential changes in modifiable habits (i.e., sleep and diet) and their effect on our outcomes.

## 5. Conclusions

Improving sleep quality may minimize cardiometabolic decline though mechanisms involving inflammation, oxidative stress, and antioxidants. Further prospective work is needed to extend our understanding of the multiple pathways that may govern these factors.

## Figures and Tables

**Table 1:** Characteristics of the US adult population ≥20 years of age.

Characteristics	Sleep quality				*p* value
	Good (*n* = 428)	Fair (*n* = 488)	Poor (*n* = 579)	Very poor (*n* = 577)	
Age (mean (95% CI))	51.5 (48.7, 54.3)	48.9 (46.2, 51.6)	46.6 (44.4, 48.8)	46.7 (44.5, 48.9)	<0.05
Age categories (% (95% CI))					
20 to <40 years	28.2 (21.7, 34.7)	33.9 (28.6, 39.2)	36.8 (31.8, 41.8)	33.7 (29.1, 38.4)	<0.05
40 to <65 years	45.9 (40.4, 51.5)	44.8 (39.2, 50.4)	47.1 (44.1, 50.1)	51.3 (45.7, 56.9)	
≥65 years	25.9 (20.3, 31.4)	21.3 (15.9, 26.6)	16.1 (12.2, 20.0)	15.0 (10.0, 19.9)	
Sex					
Men	60.8 (55, 66.6)	51.5 (46.5, 56.4)	51.1 (47.5, 54.8)	43.4 (39.5, 47.4)	<0.05
Women	39.2 (33.4, 45.0)	48.5 (43.6, 53.5)	48.9 (45.2, 52.5)	56.6 (52.6, 60.5)	
Ethnicity					
Non-Hispanic White	58.1 (49.1, 67.2)	73.8 (66.7, 80.9)	75.2 (67.4, 82.9)	73.6 (68.3, 78.9)	<0.05
Non-Hispanic Black	15.6 (10.7, 20.5)	10.0 (5.5, 14.6)	8.6 (5.1, 12.2)	13.0 (8.9, 17.1)	
Mexican American	14.8 (9.1, 20.6)	7.0 (3.5, 10.6)	6.6 (4.6, 8.6)	5.3 (3.2, 7.3)	
Others	11.4 (6.0, 16.8)	9.2 (3.6, 14.7)	9.6 (5.1, 14.1)	8.1 (5.0, 11.3)	
Education					
<High school	26.1 (18.7, 33.5)	16.5 (11.2, 21.8)	14.2 (10.9, 17.5)	16.8 (12.5, 21.1)	<0.05
High school	25.5 (19.0, 32.1)	24.9 (20.1, 29.7)	24.4 (20.2, 28.6)	27.7 (24.0, 31.4)	
College	48.4 (36.2, 60.6)	58.6 (51.5, 65.7)	61.4 (56.0, 66.8)	55.5 (50.0, 61.0)	
Income					
<$20,000	16.8 (12.0, 21.6)	14.7 (10.8, 18.6)	12.9 (9.2, 16.7)	18.8 (14.1, 23.6)	NS
$20,000–44,999	33.7 (25.8, 41.5)	33.1 (27.2, 39.0)	30.3 (23.6, 37.0)	31.5 (24.0, 39.0)	
≥$45,000	49.5 (39.5, 59.6)	52.2 (45.6, 58.9)	56.8 (49, 64.5)	49.7 (42.1, 57.3)	
Smoking					
None	52.1 (43.0, 61.3)	47.7 (40.8, 54.5)	52.9 (47.9, 58)	42.8 (36.6, 49.0)	<0.05
Current	20.7 (14.7, 26.8)	20.6 (14.9, 26.4)	22.1 (17.1, 27.2)	34.3 (29.7, 39.0)	
Past	27.1 (21.5, 32.8)	31.7 (26.8, 36.6)	24.9 (20.8, 29.0)	22.8 (19.3, 26.4)	
Alcohol intake					
0 drinks/d	36.6 (30.9, 42.3)	29.8 (23.3, 36.3)	28.1 (22.2, 34.0)	33.6 (29.4, 37.8)	NS
<3 drinks/d	41.3 (34.4, 48.3)	46.3 (39.5, 53.1)	44.8 (39.0, 50.5)	39.7 (34.5, 44.8)	
≥3 drinks/d	22.1 (16.9, 27.3)	23.9 (19.2, 28.6)	27.1 (22.4, 31.9)	26.8 (21.8, 31.7)	
Recreational physical activity					
None reported	41.2 (34.2, 48.1)	28.2 (24.2, 32.3)	28.2 (22.9, 33.5)	35.6 (31.2, 39.9)	<0.05
<500 MET·min/w	16.4 (11.3, 21.5)	22.2 (17.8, 26.6)	25.1 (20.2, 30.1)	22.4 (18.3, 26.5)	
≥500 MET·min/w	42.4 (36.9, 48.0)	49.6 (44.1, 55.1)	46.6 (41.9, 51.4)	42.1 (35.2, 49.0)	

Mean (95% CI) for continuous variables and % (95% CI) for categorical variables. Overall sleep quality was calculated based on six questions on sleep habits of participants and categorized into quartiles: good (<3), fair (≥3 to 7), poor (≥7 to 12), and very poor (≥12). *p* < 0.05, two-sided; ANOVA or Chi-square, as appropriate. NS: not significant. Sum of weights = 95,276,598.

**Table 2:** Indirect effect of mediators on the sleep quality-metabolic syndrome and sleep quality-number of MetS components relationship.

Mediator	*c*-*c*′	*ab* (95% CI_sobel_)	
	MetS	MetS	Number of MetS components
C-Reactive Protein (nM)	0.012	0.014 (−0.007, 0.035)	0.011 (−0.004, 0.026)
*γ*-Glutamyl transferase (U/L)	0.022	0.042 (−0.006, 0.090)	0.011 (−0.003, 0.025)
Bilirubin (*µ*M)	0.006	0.006 (−0.001, 0.013)	0.006 (−0.001, 0.012)
Carotenoids (*µ*M)	0.002	0.002 (−0.012, 0.016)	0.001 (−0.010, 0.013)
Uric acid (*µ*M)	−0.009	−0.011 (−0.038, 0.017)	−0.008 (−0.030, 0.013)
Vitamin A (*µ*M)	0.017	**0.016 **(0.001,0.031)^*∗*^	**0.015 **(0.001,0.028)^*∗*^
Vitamin C (*µ*M)	0.019	**0.018 **(0.003,0.034)^*∗*^	**0.014 **(0.002, ****0.026)^*∗*^
Vitamin D (nM)	−0.004	−0.005 (−0.024, 0.014)	−0.004 (−0.021, 0.013)
Vitamin E (*µ*M)	−0.015	−0.016 (−0.040, 0.008)	−0.014 (−0.035, 0.006)

MetS is metabolic syndrome. *c*-*c*′ and *ab* are indirect effect. ^*∗*^Significant for Joint test, and bold means significant for Sobel test.

**Table 3:** Indirect effect of mediators on the sleep quality-individual MetS component relationship.

Mediator	*ab* (95% CI_sobel_)					
	Waist circumference	Systolic blood pressure	Diastolic blood pressure	Triglycerides	HDL cholesterol	Fasting plasma glucose
C-Reactive Protein (nM)	0.204 (−0.004,0.412)^*∗*^	0.058 (−0.023, 0.140)	0.035 (−0.009,0.078)^*∗*^	0.005 (−0.002, 0.011)	−0.001 (−0.003, 0.000)	0.009 (−0.002,0.020)^*∗*^
*γ*-Glutamyl transferase (U/L)	0.101 (−0.025, 0.226)	0.101 (−0.037, 0.240)	0.049 (−0.013, 0.112)	0.010 (−0.004, 0.023)	−0.001 (−0.002, 0.001)	0.013 (−0.005, 0.032)
Bilirubin (*µ*M)	0.016 (−0.110, 0.142)	0.014 (−0.06, 0.088)	−0.002 (−0.012, 0.008)	0.000 (0.000, 0.000)	0.000 (−0.003, 0.002)	0.000 (−0.008, 0.007)
Carotenoids (*µ*M)	0.094 (−0.023, 0.211)	0.071 (−0.01, 0.151)	−0.010 (−0.034, 0.014)	0.001 (−0.001, 0.004)	−0.001 (−0.003, 0.000)	0.004 (−0.004, 0.012)
Uric acid (*µ*M)	−0.115 (−0.411, 0.181)	−0.051 (−0.192, 0.09)	−0.013 (−0.052, 0.027)	−0.004 (−0.016, 0.007)	0.002 (−0.003, 0.006)	0.000 (−0.003, 0.002)
Vitamin A (*µ*M)	0.046 (−0.022, 0.115)	**0.158** (0.023,0.294)^*∗*^	0.001 (−0.034, 0.037)	**0.019** (0.002,0.036)^*∗*^	0.001 (−0.001, 0.002)	0.000 (−0.005, 0.006)
Vitamin C (*µ*M)	**0.227** (0.039,0.414)^*∗*^	0.049 (−0.028, 0.125)	**0.076** (0.008,0.145)^*∗*^	**0.010** (0.001,0.020)^*∗*^	**−0.005** (−0.009, −0.001)^*∗*^	**0.011** (0.003,0.018)^*∗*^
Vitamin D (nM)	−0.057 (−0.292, 0.178)	−0.034 (−0.143, 0.074)	−0.008 (−0.040, 0.023)	−0.002 (−0.008, 0.005)	0.001 (−0.003, 0.004)	−0.004 (−0.018, 0.011)
Vitamin E (*µ*M)	−0.064 (−0.160, 0.032)	−0.099 (−0.266, 0.068)	−0.035 (−0.092, 0.022)	−0.027 (−0.067, 0.012)	0.000 (−0.001, 0.001)	−0.010 (−0.025, 0.005)

*ab* is indirect effect. ^*∗*^Significant for Joint test, and bold means significant for Sobel test.

**Table 4:** Indirect effect of mediators on the sleep quality-metabolic syndrome and sleep quality-number of MetS components relationship for men.

Mediator	*c*-*c*′	*ab* (95% CI_sobel_)	
	MetS	MetS	Number of MetS components
C-Reactive Protein (nM)	0.002	0.001 (−0.006, 0.008)	0.002 (−0.006, 0.010)
*γ*-Glutamyl transferase (U/L)	0.024	0.042 (−0.013, 0.097)	0.014 (−0.003, 0.031)
Bilirubin (*µ*M)	0.001	0.000 (−0.013, 0.013)	0.000 (−0.011, 0.011)
Carotenoids (*µ*M)	0.010	−0.014 (−0.032, 0.004)	−0.011 (−0.024, 0.002)
Uric acid (*µ*M)	0.013	−0.001 (−0.06, 0.058)	−0.001 (−0.041, 0.039)
Vitamin A (*µ*M)	0.017	0.015 (−0.004, 0.034)	**0.014 **(0.000,0.028)^*∗*^
Vitamin C (*µ*M)	0.012	0.007 (−0.021, 0.036)	0.005 (−0.016, 0.026)
Vitamin D (nM)	−0.009	−0.010 (−0.024, 0.004)	−0.011 (−0.024, 0.002)
Vitamin E (*µ*M)	−0.018	**−0.029 **(−0.057,0.000)^*∗*^	**−0.026 **(−0.050, −0.002)^*∗*^

MetS is metabolic syndrome. *ab* is indirect effect. ^*∗*^Significant for Joint test, and bold means significant for Sobel test.

**Table 5:** Indirect effect of mediators on the sleep quality-metabolic syndrome and sleep quality-number of MetS components relationship for women.

Mediators	*c*-*c*′	*ab* (95% CI_sobel_)	
	MetS	MetS	Number of MetS components
C-Reactive Protein (nM)	0.036	**0.044 **(0.011,0.078)^*∗*^	**0.035 **(0.012,0.059)^*∗*^
*γ*-Glutamyl transferase (U/L)	0.042	0.049 (−0.012,0.109)^*∗*^	0.017 (−0.001,0.035)^*∗*^
Bilirubin (*µ*M)	0.003	0.003 (−0.005, 0.011)	0.005 (−0.005, 0.015)
Carotenoids (*µ*M)	0.012	0.020 (0.000, 0.040)	0.017 (0.000, 0.033)
Uric acid (*µ*M)	0.035	**0.056 **(0.007,0.105)^*∗*^	**0.043 **(0.005,0.08)^*∗*^
Vitamin A (*µ*M)	0.022	0.025 (−0.006, 0.055)	0.021 (−0.005, 0.047)
Vitamin C (*µ*M)	0.031	**0.032 **(0.008,0.055)^*∗*^	**0.022** (0.004,0.041)^*∗*^
Vitamin D (nM)	0.006	0.013 (−0.036, 0.062)	0.010 (−0.028, 0.048)
Vitamin E (*µ*M)	−0.014	**−0.032 **(−0.062, −0.002)^*∗*^	−0.003 (−0.030, 0.024)

MetS is metabolic syndrome. *ab* is indirect effect. ^*∗*^Significant for Joint test, and bold means significant for Sobel test.

**Table 6:** Indirect effect of mediators on the sleep quality-individual MetS component relationship for men.

Mediators	*ab* (95% CI_sobel_)					
	Waist circumference	Systolic blood pressure	Diastolic blood pressure	Triglycerides	HDL cholesterol	Fasting plasma glucose
C-Reactive Protein (nM)	0.046 (−0.159, 0.251)	0.01 (−0.042, 0.062)	0.009 (−0.033, 0.051)	0.000 (−0.002, 0.003)	0.000 (−0.003, 0.002)	0.001 (−0.003, 0.006)
*γ*-Glutamyl transferase (U/L)	0.096 (−0.015, 0.206)	0.095 (−0.028, 0.218)	0.075 (−0.023, 0.173)	0.014 (−0.005, 0.033)	0.001 (−0.001, 0.002)	0.019 (−0.009, 0.047)
Bilirubin (*µ*M)	0.001 (−0.099, 0.100)	0.001 (−0.074, 0.075)	0.000 (−0.031, 0.031)	0.000 (−0.004, 0.004)	0.000 (−0.002, 0.002)	0.000 (−0.003, 0.003)
Carotenoids (*µ*M)	−0.12 (−0.264, 0.024)	−0.066 (−0.163, 0.03)	0.001 (−0.028, 0.03)	0.002 (−0.003, 0.008)	0.003 (−0.001, 0.006)	−0.006 (−0.014, 0.003)
Uric acid (*µ*M)	−0.010 (−0.535, 0.515)	−0.003 (−0.12, 0.115)	−0.004 (−0.141, 0.133)	0.000 (−0.021, 0.020)	0.000 (−0.007,0.008)^*∗*^	−0.001 (−0.012, 0.009)
Vitamin A (*µ*M)	−0.041 (−0.148, 0.067)	0.093 (−0.008,0.194)^*∗*^	0.031 (−0.032, 0.093)	**0.024** (0.002,0.045)^*∗*^	0.002 (−0.001, 0.004)	0.000 (−0.008, 0.007)
Vitamin C (*µ*M)	0.067 (−0.202, 0.336)	0.021 (−0.081, 0.123)	0.029 (−0.087, 0.144)	0.004 (−0.011, 0.019)	−0.001 (−0.007,0.004)^*∗*^	0.006 (−0.006, 0.018)
Vitamin D (nM)	−0.169 (−0.364, 0.025)	−0.048 (−0.12, 0.024)	−0.042 (−0.121, 0.037)	−0.009 (−0.020, 0.002)	0.004 (0.000,0.008)^*∗*^	−0.012 (−0.027, 0.004)
Vitamin E (*µ*M)	−0.107 (−0.228,0.013)^*∗*^	−0.114 (−0.243,0.016)^*∗*^	−0.105 (−0.204, −0.007)^*∗*^	**−0.059** (−0.115, −0.004)^*∗*^	0.001 (−0.001, 0.004)	−0.010 (−0.024, 0.004)

*ab* is indirect effect. ^*∗*^Significant for Joint test, and bold means significant for Sobel test.

**Table 7:** Indirect effect of mediators on the sleep quality-individual MetS component relationship for women.

Mediators	*ab* (95% CI_sobel_)					
	Waist circumference	Systolic blood pressure	Diastolic blood pressure	Triglycerides	HDL cholesterol	Fasting plasma glucose
C-Reactive Protein (nM)	**0.539** (0.204,0.875)^*∗*^	**0.189** (0.023,0.355)^*∗*^	0.093 (−0.005, 0.190)	0.017 (0.000,0.034)^*∗*^	−0.004 (−0.009, 0.001)	**0.030** ** (0.011, 0.048)**
*γ*-Glutamyl transferase (U/L)	**0.143** (−0.031,0.317)^*∗*^	0.188 (−0.006, 0.382)	0.031 (−0.014, 0.076)	0.011 (−0.004, 0.026)	0.000 (−0.002, 0.002)	0.015 (−0.004, 0.034)
Bilirubin (*µ*M)	0.051 (−0.059, 0.161)	0.002 (−0.06, 0.063)	0.018 (−0.025, 0.060)	0.004 (−0.004, 0.012)	−0.002 (−0.006,0.002)^*∗*^	−0.002 (−0.010, 0.007)
Carotenoids (*µ*M)	**0.199** **(0.001, 0.397)**	0.133 (−0.005,0.272)^*∗*^	−0.019 (−0.059, 0.022)	0.003 (−0.001, 0.008)	−0.004 (−0.009, 0.000)	0.012 (−0.002, 0.025)
Uric acid (*µ*M)	**0.474** (0.058,0.890)^*∗*^	**0.359** (0.065,0.654)^*∗*^	−0.022 (−0.075, 0.032)	**0.016** (0.002,0.031)^*∗*^	−0.003 (−0.007,0.000)^*∗*^	**0.013** (−0.002,0.028)^*∗*^
Vitamin A (*µ*M)	0.073 (−0.044, 0.191)	0.276 (−0.049, 0.602)	−0.052 (−0.145, 0.042)	0.022 (−0.005, 0.048)	0.004 (−0.001, 0.009)	0.000 (−0.008, 0.009)
Vitamin C (*µ*M)	**0.445** (0.162,0.728)^*∗*^	0.024 (−0.199, 0.246)	**0.127** (0.020,0.234)^*∗*^	**0.014** (0.003,0.025)^*∗*^	**−0.007** ** (−0.013, −0.002)**	**0.017** (0.001,0.034)^*∗*^
Vitamin D (nM)	0.140 (−0.373, 0.654)	0.054 (−0.234, 0.342)	0.006 (−0.027, 0.039)	0.002 (−0.006, 0.011)	−0.002 (−0.007,0.004)^*∗*^	0.004 (−0.020, 0.028)
Vitamin E (*µ*M)	−0.015 (−0.154, 0.123)	−0.006 (−0.327, 0.315)	−0.001 (−0.034, 0.033)	−0.005 (−0.046, 0.037)	0.000 (−0.001, 0.001)	−0.009 (−0.040, 0.022)

*ab* is indirect effect. ^*∗*^Significant for Joint test, and bold means significant for Sobel test.
